# Insect-Like Organization of the Stomatopod Central Complex: Functional and Phylogenetic Implications

**DOI:** 10.3389/fnbeh.2017.00012

**Published:** 2017-02-07

**Authors:** Hanne H. Thoen, Justin Marshall, Gabriella H. Wolff, Nicholas J. Strausfeld

**Affiliations:** ^1^Sensory Neurobiology Group, Queensland Brain Institute, University of Queensland, St. LuciaBrisbane, QLD, Australia; ^2^Department of Biology, University of WashingtonSeattle, WA, USA; ^3^Department of Neuroscience, School of Mind, Brain and Behavior, University of ArizonaTucson, AZ, USA

**Keywords:** central complex, stomatopod, crustaceans, insects, eye movements, evolution

## Abstract

One approach to investigating functional attributes of the central complex is to relate its various elaborations to pancrustacean phylogeny, to taxon-specific behavioral repertoires and ecological settings. Here we review morphological similarities between the central complex of stomatopod crustaceans and the central complex of dicondylic insects. We discuss whether their central complexes possess comparable functional properties, despite the phyletic distance separating these taxa, with mantis shrimp (Stomatopoda) belonging to the basal branch of Eumalacostraca. Stomatopods possess the most elaborate visual receptor system in nature and display a fascinating behavioral repertoire, including refined appendicular dexterity such as independently moving eyestalks. They are also unparalleled in their ability to maneuver during both swimming and substrate locomotion. Like other pancrustaceans, stomatopods possess a set of midline neuropils, called the central complex, which in dicondylic insects have been shown to mediate the selection of motor actions for a range of behaviors. As in dicondylic insects, the stomatopod central complex comprises a modular protocerebral bridge (PB) supplying decussating axons to a scalloped fan-shaped body (FB) and its accompanying ellipsoid body (EB), which is linked to a set of paired noduli and other recognized satellite regions. We consider the functional implications of these attributes in the context of stomatopod behaviors, particularly of their eyestalks that can move independently or conjointly depending on the visual scene.

## Introduction

### Stomatopod Vision and Midbrain Organization

**Figure 1:**
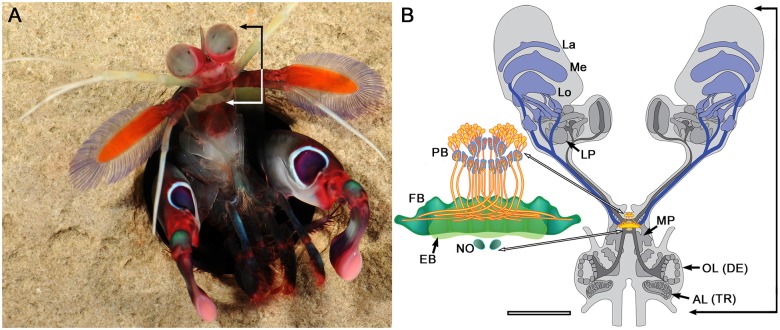
**Stomatopod crustacean, brain and central complex. (A)** *Gonodactylus smithii*, with raised eyes and frontal “head” region (bracketed). Image: Roy Caldwell. **(B)** Schematic of brain (based on sections of *Neogonodactylus oerstedii*) showing the fused neuromeres of tritocerebrum (indicated by the antennal lobes, AL (TR)), deutocerebrum (indicated by the antennular olfactory lobes, OL (DE)) and the medial protocerebrum (MP), the latter containing the central complex (enlarged showing protocerebral bridge, PB; fan-shaped and ellipsoid bodies, FB, EB; noduli, NO). Neuropils within the eyestalks comprise the lateral protocerebrum (LP) and optic lobes (La, Me, Lo). Ascending axon bundles (dark gray) from the OL extend to the lateral protocerebra; descending axon bundles (dark blue) extend from the optic lobes, and optic glomeruli to reach the midbrain and central complex. Scale bar for a small example of this species is 2 mm.

Mantis shrimp (Stomatopods) are a group of stemward eumalacostracans that separated from other malacostracan lineages about 400 million years ago (Schram, 1969). They possess one of the most elaborate visual systems known, at least at the receptor level (Marshall, 1988; Cronin and Marshall, 1989a,b; Marshall et al., 2007). An equatorial system of photoreceptors (called the midband) can detect up to 12 different spectral channels (Marshall et al., 1991b, 1996; Cronin et al., 1994), as well as both linear (Marshall et al., 1991a, 1999; Kleinlogel and Marshall, 2006) and circular polarized light (Chiou et al., 2008; Gagnon et al., 2015). The midband divides the eye into three different regions: the midband itself, and the dorsal and ventral hemispheres, some combination of which must also mediate luminance and spatial vision tasks. The retina, together with the nested optic neuropils and numerous discrete neuropils comprising the lateral protocerebrum (LP) are contained within the distally expanded mobile eyestalk. The optic neuropils and LP are further connected to the midbrain by axon bundles that project to it through the eyestalks (Figure 1).

One unique feature of stomatopods is that they are able to move their eyestalks independently and asymmetrically (Land et al., 1990) using coordinated actions of eight independent muscles (Jones, 1994). These movements include slow “scanning” movements and fast “saccadic” movements (Land et al., 1990; Marshall et al., 2014) as well as object tracking and optomotor stabilizations (Cronin et al., 1988, 1991) mediated by pitch, yaw and roll rotations of the eye. Some of these movements are thought to be involved in optimizing visual perception of certain modalities such as polarized light (Daly et al., 2016) and are possibly also involved in their putative interval-decoding color vision system where the perceived color corresponds to the peak sensitivity of the most responsive photoreceptor (Thoen et al., 2014; Zaidi et al., 2014). Another unique feature is that each eye has the potential for stereopsis due to the overlapping visual fields of the convex upper and lower eye halves (Marshall and Land, 1993). Observing the independent eye movements of stomatopods gives the strong impression of a crustacean equipped with two independent brains that occasionally function in unison (for example see Bok, 2012).

Stomatopods can switch from asymmetric to more coordinated eye movements, which appear to be triggered by threshold-sized objects (such as potential prey, predators, competitors or mates) detected by one or both eyes. This switch and subsequent actions are likely to be mediated by circuits that are supplied by inputs from both eyestalks. Hundreds of axons extend from each LP, many of which distribute to lateralized neuropils in the mid-brain. However, as demonstrated by dye fills (see below) certain axons converge at a system of midline neuropils known as the central complex (CX). In dicondylic insects the CX is implicated in the selection and execution of motor actions (Martin et al., 2015), and all species thus far examined have the same ground pattern organization (Williams, 1975; Strausfeld, 1976; Hanesch et al., 1989). Homologous but divergent centers are found in Myriapoda, Chelicerata and Onychophora (Loesel et al., 2002; Strausfeld et al., 2016). Comparable midline neuropils found in vertebrates (Strausfeld and Hirth, 2013), polychaete annelids (Heuer et al., 2010) and polyclad flatworms (Wolff and Strausfeld, 2015) suggest an ancient Precambrian origin for this center.

Stomatopoda is prominent amongst crustacean orders in that its central complex is organized much like that of homologous centers in dicondylic insects. Furthermore, many stomatopod species display highly coordinated appendicular actions, in addition to independent and conjoint movements of the eyestalks. We have consequently begun a wide-ranging investigation of the stomatopod CX in terms of its functional organization and control properties, comparing these with the CX of dicondylic insects. Here we discuss the first phase of this study showing that the stomatopod CX possesses structural characters that show multiple correspondences to structures in the CX of Dicondylia. We further explore the functional implications of these correspondences and suggest likely roles of the CX in relation to the stomatopods behavioral repertoire.

## Materials and Methods

### Animals

Between 4 and 50 Stomatopods of the species *Pseudosquilla ciliata, Gonodactylus smithii* and *Haptosquilla trispinosa* were obtained from designated areas overseen by the Lizard Island Research Station, Australia (GBRMPA Permit no. G12/35005.1, Fisheries Act no. 140763). Seventy two *Neogonodactylus oerstedii* were collected in the Florida Keys, USA. Twelve specimens of *Ligia exotica* were collected from a beach near Qingdao Pier, Qingdao, China.

### Reduced Silver Staining

Using Bodian’s (1936) original method, tissue was fixed in AAF (16 ml 80% ethanol, 1 ml glacial acetic acid, 3 ml 37% formaldehyde), before being dehydrated in ascending alcohols, cleared in terpineol and embedded in Paraplast Plus (Sherwood Medical, St. Louis, MO, USA). The 12 μm serial sections were mounted on slides using albumin, then deparaffinized, rehydrated and incubated in a solution of 2.5 g Protargol (Roques, Paris, France) and 5 g copper in 250 ml double-distilled water at 60°C overnight. The sections were next washed briefly in distilled water, processed through a solution of 1% hydroquinone and 5% sodium sulfite (5 min), 1% gold chloride (9 min), 2% oxalic acid (5 min) and 5% sodium thiosulfate (5 min). Sections were dehydrated before being mounted in Entellan (Merck, Darmstadt, Germany) under coverslips.

### Immunocytochemistry

A range of different antibodies was employed to visualize structures in the stomatopod central complex (Table 1). A monoclonal antibody against synapsin (3C11, “SYNORF1”), a protein associated with synaptic vesicles in *Drosophila* (kindly provided by E. Buchner, University of Würzburg, Germany) was used at a concentration of 1:50. It has consistently been used to label brain structure in all major malacostracan subgroups, including stomatopods (Sullivan and Beltz, 2004) and recognizes at least four synapsin isoforms (70, 74, 80 and 143 kDa; Klagges et al., 1996). An antibody against serotonin (5HT, Immunostar, Hudson, WI, USA) has been previously used to label neurons in several crustacean species, including the stomatopod *Neogonodactylus oerstedii* (Derby et al., 2003) and was used at a concentration of 1:1000. A monoclonal antiserum against α-tubulin (12G10) was used at a concentration of 1:100 and was developed by Drs. J. Frankel and E. M. Nelsen. This antiserum was obtained from the Developmental Studies Hybridoma Bank developed under the auspices of the NICHD and maintained by the Department of Biology, University of Iowa (Iowa City, IA, USA). Anti-DC0, a generous gift of Dr. D. Kalderon (Columbia University, New York, NY, USA) was used at a concentration of 1:250 and recognizes the catalytic subunit of cAMP dependent protein kinase A across all arthropods investigated thus far (Wolff and Strausfeld, 2015). Antisera against FMRFamide were generously provided by Dr. E. Marder (Brandeis University, Waltham, MA, USA) and used at a concentration of 1:100. Anti-NPF antisera were generously donated by Dr. P. Shen (University of Georgia) and used at a concentration of 1:1000. Anti-GABA (Sigma-Aldrich, A2052) was used at a concentration of 1:200. Finally, cell nuclei were labeled using a blue-fluorescent DNA-stain (DAPI, Molecular Probes, D1306) or a green fluorescent nucleic acid stain (SYTO 13, Molecular Probes, S7575).

**Table 1:** **Antibody data**.

Antibody	Immunogen	Supplier	Host
Synapsin “SYNORF 1”	A glutathione S–transferase-fusion protein including a portion of a *Drosophila* synapsin homolog	DSHB, # 3C11	Mouse (monoclonal)
Alpha-tubulin	Alpha-tubulin from a mixture of *Tetrahymena thermophila* and *Tetrahymena pyriformis*	DSHB, #12G10	Mouse (monocolonal)
Serotonin (5HT)	Serotonin coupled to bovine serum albumin (BSA) with paraformaldehyde	Immunostar, Hudson, WI 20080	Rabbit, (polyclonal)
FMRFamide (671)	FMRFamide conjugated to succinylated thyroglobulin	Dr. E. Marder	Rabbit (polyclonal)
Neuropeptide F	A peptide with 36 residues synthesized based on the deduced sequence of *Drosophila* NPF and amidated at the C-terminus	Dr. P. Shen	Rabbit (polyclonal)
GABA	GABA coupled to BSA	Sigma-Aldrich, # A2052	Rabbit (polyclonal)
DC0	Purified DC0, the major catalytic subunit of *Drosophila* c-AMP-dependent protein kinase A	Dr. D. Kalderon	Rabbit (polyclonal)

#### Procedure

Animals were cold anesthetized, decapitated and dissected out in cold (4°C) fixative (4% paraformaldehyde and 10% sucrose in phosphate-buffered saline, pH 7.4 (PBS, Sigma, St. Louis, MO, USA)). Brains fixed for the synapsin and serotonergic staining was left in fixative overnight (4°C), while brains fixed for the remaining antibodies were fixed in a microwave at 18°C for two cycles of 2 min with power and 2 min under vacuum before being placed in fresh fixative overnight at 4°C. The next day the brains were washed 3× 10 min in PBS and embedded in 5% LMP agarose (LMP, A9414, Sigma Aldrich; for the synapsin and serotonergic staining) or albumin gelatin (for the remaining antibodies) before being cut at 60–150 μm thick sections using a Leica vibratome. Sections were next washed 6 × 20 min in 0.1 M PBS containing 0.5% Triton X-100 before being preincubated in 0.1 M PBS with 0.2% Triton X-100 and 5% Normal Goat Serum (NGS, Life-Technologies, Carlsbad, CA, USA 50-062Z) for 1 h at room temperature. Sections were then incubated with the respective antibodies at the concentrations listed above in either 0.1 M PBS with 0.2% Triton X-100 and 2% NGS for 3 days at 4°C (synapsin and serotonin) or with 0.1 M PBS with 0.5% Triton X-100 and 5% NGS overnight on a shaker in room temperature (for the remaining antibodies).

Sections were next rinsed for 5× 10 min in 0.1 M PBS before two different procedures were carried out. For the synapsin and serotonergic staining: incubation in 0.1 M PBS with 1% NGS containing Alexa Fluor 647 goat anti-mouse (1:250 Molecular Probes, A21236) and Alexa Fluor 568 goat anti-rabbit (1:250 Molecular Probes, A11011) for 2 h in room temperature. After rinsing in 0.1 M PBS 2 × 10 min, sections were incubated with 300 μM DAPI (Molecular probes, D1306) for 5 min, rinsed again in 0.1 M PBS for 10 min before being mounted on slides in 80% glycerol. For the remaining antibodies: 1000 μL aliquots of PBS-TX were placed in tubes with 0.25% secondary Cy2-, Cy3-, or Cy5-conjugated IgGs (Jackson ImmunoResearch, West Grove, PA, USA) and centrifuged at 13,000 rpm for 15 min at 4°C. The top 900-μL of this solution was added to each well. The well plate was left on a shaker to gently agitate the sections overnight at room temperature. Tissue sections were then washed in PBS six times over 3 h, embedded on glass slides in a medium of 25% polyvinyl alcohol, 25% glycerol and 50% PBS, and then imaged on the confocal microscope. Where applicable, sections were incubated in the fluorescent nuclear stain Syto-13 at a concentration of 1:4000 prior to embedding on glass slides.

### Whole Mount Immunocytology

Some brains were processed as whole mounts, rather than being sectioned, using the same fixation procedure as described above. After fixation, the brains were washed for 6× 10 min in 0.1 M PBS before being pre-incubated in 0.1 M PBS with 0.1% Triton X-100 and 2% NGS for 3 h in room temperature. The brains were then incubated for 6 days at 4°C in SYNORF1 diluted with 0.1 M PBS with 0.2# Triton X-100 and 2% NGS. The brains were then washed 5× 20 min in 0.1 M PBS before being incubated for 4 days in 1:250 Alexa Fluor 647 goat anti-mouse (Molecular Probes, A21236) in 0.1 M PBS with 1% NGS at 4°C. The brains were then washed in 0.1 M PBS for 3× 20 min, dehydrated in ascending series of ethanol, cleared in methyl salicylate and mounted in Permount using spacer rings to avoid tissue compression.

### Mass Filling of Neurons

Mass fills of neurons were carried out using the method of Ehmer and Gronenberg (2002). Briefly, crystals of dextran conjugated with either Texas Red (D-3328) or Fluorescein (D-3306 Molecular probes, Life Technologies) were made into a paste on a glass slide using water from condensation built up from ice placed beneath the slide. A small droplet of paste was applied to the tip of a glass electrode and inserted into either the LP or central complex. Dye was allowed to spread for about 6 h before the animal was euthanized, nervous tissue exposed and placed in fixative (4% paraformaldehyde) overnight. Dissected tissue was embedded in 5% agarose (LMP, A9414, Sigma Aldrich), vibratome sectioned at 100 or 150 μm, mounted and coverslipped using 80% glycerol.

### Image Acquisition and Processing

Sections and whole mounts labeled with antibodies against serotonin and synapsin were imaged using an LSM 710 inverted point-scanning laser confocal microscope (ARC LIEF grant no. LE130100078) with the 10× (0.45) air objective at 1024 × 1024 resolution and 0.5–1 μm depth. Sections labeled with antibodies against alpha-tubulin, FMRFamide, Neuropeptide F, GABA or DC0 were imaged using an LSM 5 Pascal confocal microscope (Zeiss, Oberkochen, Germany) with the 10× (0.45) air objective or 20× /0.5 plan Neofluar objective at 1024 × 1024 resolution and 0.5–1 μm depth. Maximum projection images were made using the *z*-project plugin in the open source software Fiji (Schindelin et al., 2012). 3D-reconstructions of the synapsin-stained tissue were created using the TrakEM-2 plugin in Fiji and visualized using the 3D-viewer. Light microscopy images of serially sectioned Bodian-stained brains were acquired using a 40× plan-apochromatic objective, employing step focusing at 1 μm increments to obtain stacks used for reconstruction. Images were adjusted for brightness, contrast, and threshold using Adobe Photoshop CC.

### Reconstructions

**Figure 2:**
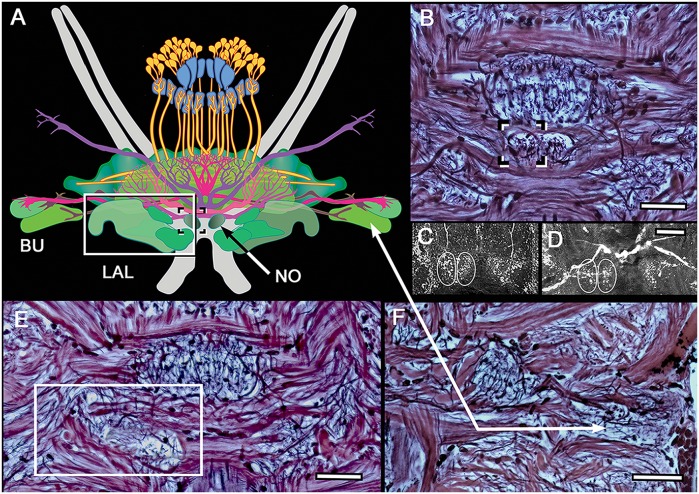
**Noduli and lateral accessory lobe (LAL) neuropils. (A)** Overview of the two lateral bulbs (Bu), paired noduli (No) and LAL. **(B)** The noduli (one boxed) are prominent and have been resolved in all species so far examined. Unlike in insects, each nodulus appears to have two side-by-side domains, as revealed by FMRF and 5HT immunocytology of *N. oerstedii* **(C)** and *H. trispinosa* **(D)**. **(E)** Bodian staining resolves the LAL as a multi-lobed neuropil, one of which (boxed) is shown here. **(F)** Bodian staining of the lateral bulbs distinguishes their large dendritic trees and different staining densities. Scale bars: **(B–F)** 50 μm.

Reconstructions of central complexes and their satellite neuropils are derived from aligned serial sections stained by the Bodian method, in which neuropils, large axons and axon bundles are delineated. Regions in successive sections are montaged for clarity, as in the case of the noduli and lateral accessory lobes (LAL; Figure 2). Additional data for reconstructions are derived from selective neuron labeling using antibodies listed in Table 1.

## Results

Before describing central complex organization in stomatopods, it is useful to briefly review here the occurrence and known attributes of these centers in insects and neuronal arrangements in the CX of crustaceans generally.

### An Outline of the Insect Central Complex

The class Insecta consists of two clades: Monocondylia and Dicondylia, the first represented by wingless Archaeognatha dating back 420 million years to the Devonian period (Labandeira et al., 1988). The second clade includes all other insects. The archaeognathan central complex is notable for its simplicity and its similarity to that of many malacostracans, with the exception of stomatopods. The archaeognathan CX consists of a bilayered spindle-shaped central body (CB) supplied by an incomplete decussation of axons from small, paired centers situated at the extreme rostromedial margins of the protocerebral lobes. These are referred to as the protocerebral bridge (PB) because the two centers are linked by axons that extend across the protocerebrum’s midline (Strausfeld, 2012). Neurons link the CB to flanking neuropils that may correspond to the LAL recognized in Dicondylia. There are, however, no associated ball-like centers, which in Dicondylia are referred to as the “noduli.”

The central complex in dicondylic insects consists of four delineated neuropils (Williams, 1975; Strausfeld, 1976, 1999; Homberg, 1985, 2008; Mobbs, 1985). The PB is usually a long and narrow bilateral neuropil extending between the most rostro-ventral medial lobes of the protocerebrum. Fully decussating axons project from the PB to a scallop-shaped multilayered neuropil called the fan-shaped body (FB) linked by through-going axons to the deeper ellipsoid body (EB), a name coined for its toroidal appearance in *Drosophila* (Power, 1943), although it is derived from an ancestrally shallow arch-like geometry typical of most Dicondylia. In pterygote (winged) insects, paired ball-like noduli ventral to the CB are reciprocally connected to the FB and EB. The dicondylic CX is subdivided into modules that repeat across the midline (Ito et al., 2014). Its PB is divided into 16–18 modules (8–9 on each side of the midline) that supply the FB through four pairs of fiber bundles termed the *w-, x-, y-*, and *z-tracts* (Williams, 1975). The modules each side of the midline are mapped point-for-point across the entire extent of the FB thereby dividing it into eight modules (four in each half) that are horizontally stratified by tangentially arranged terminals and dendrites. The noduli do not show any columnar organization, but consist of several stacked subunits. The FB and EB are flanked by the LAL that are partitioned into at least three domains, each receiving the terminals of modular neurons originating from cell bodies above the PB. The LAL is further linked to neuropils, into which premotor descending neurons extend axon collaterals. Thus, relays from the CX to the LAL, and thence to subsequent stations, are thought to gate the downstream activity of descending neurons supplying segmentally arranged sensory-motor circuits in thoracic ganglia (Namiki and Kanzaki, 2016).

In addition to the LAL, a satellite system associated with the EB referred to as the lateral complex, consists of three centers: the bulb, gall and wedge (Iwano et al., 2010; Ito et al., 2014). The CX, LAL and its satellites receive indirect afferents from higher order protocerebral neuropils, including indirect channels from the iconic mushroom bodies via relays to local interneurons in the superior medial protocerebrum (MP; Wolff and Strausfeld, 2016). From there, afferent neurons terminate across layers of the FB (Phillips-Portillo and Strausfeld, 2012; Strausfeld, 2012). With few exceptions, visual inputs to the CX are indirect, relayed to it via the lateral complex (Pfeiffer and Homberg, 2015; Held et al., 2016) and LP (Liu et al., 2006). Exceptions are connections between the PB of the locust *Schistocerca gregaria* and its anterior optic tubercle, an optic glomerulus receiving afferents from the medulla and lobula, and from the optic glomerular complex to the PB in muscomorphous Diptera (Phillips-Portillo, 2012) and from a corresponding region of the LP in *Drosophila melanogaster* (Lin et al., 2013). A direct projection into the CX, extending directly from the optic lobes, has been documented in Orthoptera (Honegger and Schürmann, 1975).

### Roles Ascribed to the Insect Central Complex

Properties of the dicondylic CX have been much debated in recent years, with the emergence of two potentially related views of its role in behaviors. One is that because the distribution of celestial e-vectors are so precisely represented in modules of the PB and other levels of the CX, the primary role of the CX is to mediate compass-like celestial navigation (Heinze and Homberg, 2007; Homberg et al., 2011; Pfeiffer and Homberg, 2015). The other is that the CX processes a dynamic representation of information about an insect’s orientation with respect to broader features of its visual surrounding relevant for path integration (Neuser et al., 2008; Triphan et al., 2010; Webb and Wystrach, 2016). While there is thus far no conclusive behavioral experimental evidence to support the CX as mediating path integration, there is compelling behavioral and optogenetic support for the CX’s role in visual action selection and landmark orientation by walking flies (Seelig and Jayaraman, 2013, 2015), as well as visual place memory (Liu et al., 2006; Neuser et al., 2008; Ofstad et al., 2011). Other functions suggested to rely on the central complex are the control of song production (Kunst et al., 2011); the control of appendicular movements requiring asymmetric actions (Bausenwein et al., 1986; Strauss and Heisenberg, 1993; Ilius et al., 2007); and the selection of behavioral actions and motor commands (Huber, 1959, 1960; Ridgel et al., 2007; Bender et al., 2010; Ritzmann et al., 2012; Guo and Ritzmann, 2013; Martin et al., 2015). The recognition of such a variety of functional roles are in part a consequence of the species studied and what each offers in terms of experimental access and the application of a palette of sensory stimuli. One emerging consensus is that the CX receives direct and highly synthesized inputs involving most sensory modalities, and that these inputs provide information from which the CX determines what motor actions are appropriate for current environmental conditions (Strausfeld and Hirth, 2013; Fiore et al., 2015).

There is agreement that modular organization of the PB and its extensions into the FB reflect a representation of the multisensory surround and thus spatially segmented sensory information. In locusts, for example, the PB carries a topographical representation of zenithal e-vector orientations (Heinze and Homberg, 2007). In cockroaches, the locations of haptic mechanosensory stimuli are represented across the PB (Ritzmann et al., 2008) as are the representation of directional motion in flies (Phillips-Portillo, 2012). It is likely that acoustic and other mechanical stimuli are characterized as representations in overlaying sensory space.

There is, however, a second important correlate of modularity; not in the PB but in the FB where the more defined its modular subunits, the more these indicate the ability of the species to execute highly coordinated appendicular actions: tasks such as climbing or obstacle avoidance that require asymmetric but coordinated multijoint actions (Strausfeld and Hirth, 2013).

### Overview of the Stomatopod Brain

Stomatopods have bulbous eyestalks that contain the four nested optic lobe neuropils serving the compound eye together with the neuropils of the LP (Figure 1). The LP is elaborate, comprising neuropils that are obvious homologs of those in other eumalacostracans in addition to centers that appear to be unique to Stomatopoda. Optic glomeruli are numerous. Preliminary observations show these connected by many discrete tracts to other regions of the LP. Several axon bundles extend through the eyestalks that connect lateral protocerebral neuropils, in addition to the medulla and lobula, to the midbrain. A substantial volume of each eyestalk is also occupied by the olfactory globular tract, which originates in the deutocerebrum’s olfactory lobes (OL) and extends out to the lateral protocerebra (Figure 1). The central brain comprises the fused trito-, deuto- and the medial regions of protocerebral ganglia. The central complex is situated towards the rostrum and consists of a well-defined PB, its projections to the “CB” (the FB and the EB), paired noduli, lateral accessary lobes and an accessory complex (Figures 1, 2). These combined features typify the central complex of dicondylic insects, but within Crustacea, appear to be unique to stomatopods.

### The Stomatopod Central Complex

The stomatopod central complex features a prominent PB that supplies decussating axons to a two component CB: a broad tapering upper division (here named the FB) and a narrower lower division (the EB) that provides axons to a pair of defined noduli (Figure 1). Lateral to and some distance from the CB are two clearly defined neuropils connected by axons to the EB. The disposition and connections of these neuropils correspond to the dicondylic lateral and medial bulbs and are distinct from the paired LAL that lie ventral to the EB (Figure 2).

**Figure 3:**
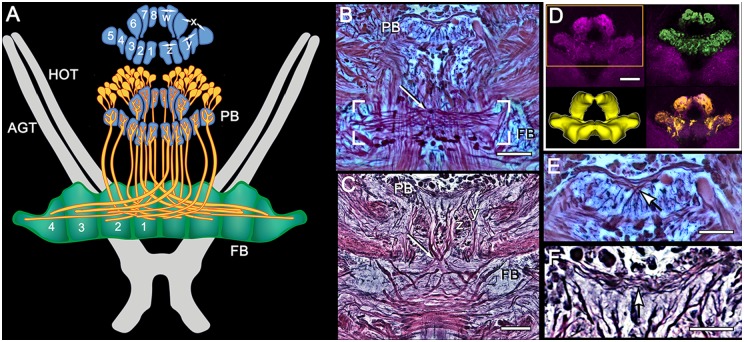
**Modular organization of the PB and projections to the FB. (A)** Reconstruction from Bodian serial sections (*Pseudosquilla ciliata*) and synapsin immunocytology (*Gonodactylus smithii*) resolve eight modules of the PB. Each module is numbered 1–8. Pairs of modules relate to the *w, x, y, z* ground pattern of axon projections originally described for the insect CX. Each PB module provides bundled axons (each schematized as a single fiber) that map all eight modules from each side of the bridge across the entire FB, itself divided into eight modules. **(B)** Bodian stained decussation (box) and PB in *P. ciliata*. Region of decussation arrowed. **(C)** Bodian-stained decussation (arrowed) in *G. smithii* where the *y* and *z* bundles are clearly resolved. **(D)** Anti-synapsin labeled PBs of* G. smithii*. The box indicates the volume used for the reconstruction lower left. The top- and bottom-right panels show feature extractions revealing tangential processes extending across the bridge (green profiles) and some of the modular dendritic arrays of modular neurons supplying the FB (yellow profiles). **(E,F)** Enlargements showing tangential processes extending across the bridge. As in other pancrustaceans, these characteristically invert their top-down order at the midline (arrowed). Scale bars: **(B–D)** 50 μm: **(E,F)** 25 μm.

**Figure 4:**
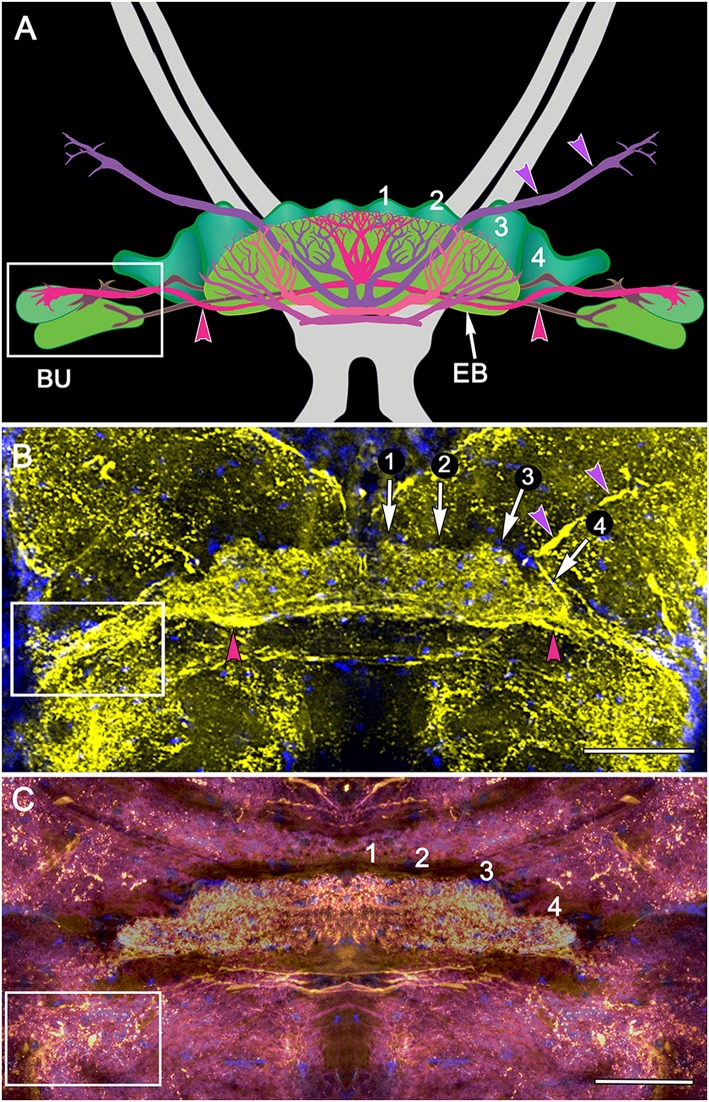
**Organization of modules and fan-shaped neurons. (A)** Reconstruction of the FB and EB of *P. ciliata*, and some of its largest fan-shaped neurons. These originate from the lateral bulbs (Bu) and anterolateral protocerebral neuropils. **(B,C)** Serotonin immunolabeling resolved the modular organization of the FB (1–4) as well as major axons, some of which correspond to those identified in *P. ciliata*. Of interest are minor differences of serotonergic labeling in these two species (*H. trispinosa* in **B**, *G. smithii* in **C**), particularly the density of labeling and the stratification of the FB, which in *G. smithii* clearly resolves three layers. The boxed areas indicate the neuropil of the bulbs. Scale bars: **(B,C)** 100 μm.

**Figure 5:**
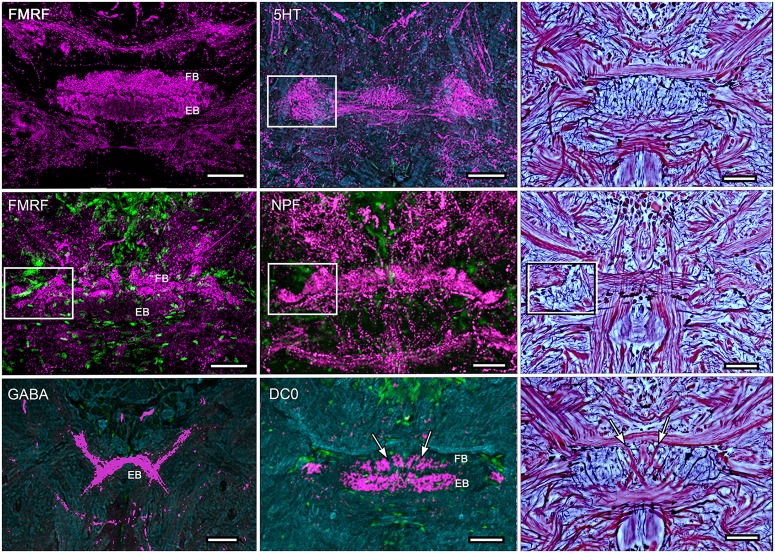
**Immunocytological partitions of the fan-shaped and ellipsoid bodies in *Neogonodactylus oerstedii*.** Antibodies raised against FMRFamide resolve the EB (upper left) and the upper layer of the modular FB (middle left). In contrast, anti-5HT labels modules through the depth of the FB. Anti-NPF also selectively resolves modules in the FB. In contrast anti-GABA thus far resolves an arch-like territory in the most ventral area of the EB that appears to be supplied by axons entering it from the anterior protocerebrum. Anti-DC0 labels the EB, as it does in *Coenobita clypeatus* and dicondylic insects (Wolff et al., 2012). Bodian-stained CXs (right hand column) show corresponding cytoarchitectures in *P. ciliata*. Abbreviations as for other figures. Corresponding areas shown boxed, corresponding axon trajectories indicated by arrows. Scale bars, 50 μm.

The architecture of the stomatopod PB (Figure 3) appears to be more elaborate than in other crustaceans and, possibly, in Dicondylia. Located at the extreme rostral margin of the brain, it extends as one continuous neuropil linking both protocerebral hemispheres. This part of the bridge is composed of 6–8 modules on either side of the midline, each of which provides bundled axons that project to the CB. The bridge has a further heterolateral extension from its dorsal side that carries a system of decussating axons between the two protocerebral hemispheres. Synapsin labeling reveals two protruding branches originating from the front of the bridge that extend a short distance rostrally before bending towards the dorsal surface of the protocerebral lobes. These branches are of a similar thickness to the bridge itself, and both Bodian and synapsin-labeled preparations show these to be connected to the bridge (Figure 3). Axons extending from each side of the PB cross each other above their entry into the CB and then extend laterally to overlap each other in the FB itself. Anti-5HT immunolabeling separately distinguishes the upper and lower neuropils of the FB (Figure 4) and also resolves a third layer with fiber bundles extending laterally on the proximal side of the EB. Other antibodies, such as anti-DC0 (Figure 5), reveal the correspondence of the FB and EB to the same named centers in Dicondylia. Anti-5HT, -Neuropeptide F and -FMRF all reveal the FB as comprising eight modules, four each side of the midline (Figures 4, 5). Anti-5HT labeling also resolves large fan-shaped tangentials with small branches in both the FB and EB, and large axons extending from the lateral and medial bulb (Figure 4). Neuropeptide F shows some labeling of the decussating neurons from the PB (Figure 5). Antisera against GABA show labeling of an arch-like territory in the EB, whereas DC0 mainly resolves the EB. However, we consider the results of GABA immunocytology still incomplete using the present antiserum as it strongly labels numerous neuronal perikarya but resolves very few processes. Figure 2 illustrates the paired noduli attached to the ventral-proximal side of the EB. The noduli appear ovoid, comprising two adjacent synaptic territories.

**Figure 6:**
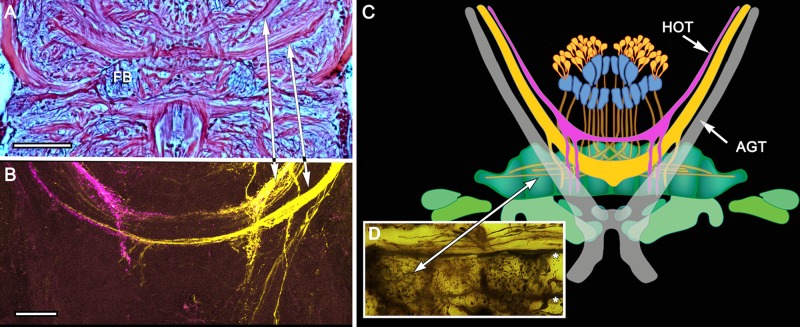
**Eyestalk convergence at the central complex. (A)** Silver stained brain of *P. ciliata* reveals numerous heterolateral fiber projections to the central complex amongst which are tracts originating from the eyestalks (arrowed). **(B)** Dye tracing in *Haptosquilla trispinosa* resolves tracts as providing processes mainly to the FB, showing that most but not all fibers appear to terminate there. **(C)** Summary figure showing FB in relation to the antennal glomerular tract (AGT), carrying olfactory neuron relays, and the two main tributaries of the heterolateral optic tracts (HOT). **(D)** Golgi impregnation showing eyestalk axons (above asterisks) extending across the CX, providing discrete terminal processes clustered in the FB modules. Scale bars: **(A)** 50 μm; **(B)** 100 μm.

**Figure 7:**
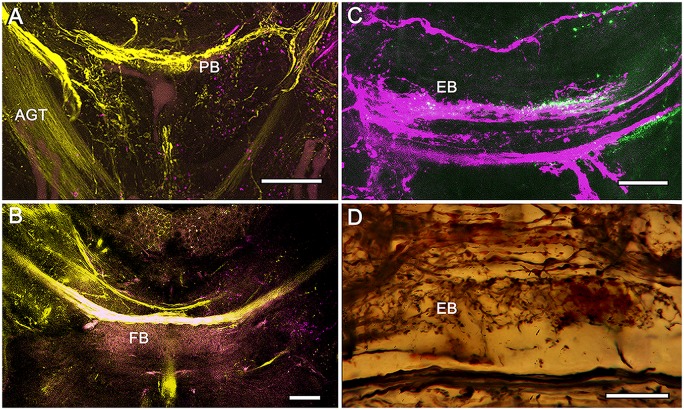
**Eyestalk convergence at the central complex. (A)** Dextran-fluorescein fills into the LP retrogradely label axons in the AGT and anterogradely filled axons extending to lateral midbrain regions as well as the midline PB. **(B)** Large heterolateral axons from the LP supplying the FB, their upper margins delineating FB modules. **(C)** Dextran-Texas Red fills reveal heterolateral inputs to the EB. **(D)** Detail of the EB showing heterolateral terminals of eyestalk axons. Scale bars: **(A,B)** 50 μm; **(C,D)** 25 μm.

Although much of the internal organization of the stomatopod CX is at present unknown, there is compelling evidence that efferents from the LP, including its visual neuropils, converge in CX neuropils. Injections of tracer dyes into lateral protocerebral neuropils fill numerous axons that project through the eyestalks into the midbrain. While many of these terminate in lateral neuropils, a number of others converge at the CX providing it with terminal arborizations (Figures 6, 7). Certain of these are clearly constrained within modules of the FB (Figure 6D); others extend diffusely across the width of the EB (Figure 7D). Terminals arranged across the PB (Figure 7A) are reminiscent of optic lobe inputs to the PB identified in Diptera (Phillips-Portillo, 2012; Lin et al., 2013).

## Discussion

### The Central Complex of Crustaceans Other than Stomatopoda

**Figure 8:**
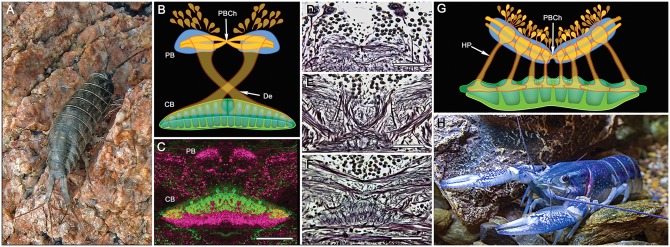
**PB and central body (CB) connections in two other eumalacostracans. (A)** The littoral isopod *Ligia exotica*. **(B)** Reconstruction of the PB and bistratified CB showing the incomplete decussation of axons from each side of the PB to the opposite site of the CB. Like other pancrustaceans, transverse fibers spanning the PB undergo a chiasma-like cross over at the mid-line (PBCh). **(C)** In many eumalacostracans, the CX lacks noduli and an obvious EB homolog. Instead, the composite CB is clearly divided into two levels each with different immunocytological properties: here affinities to allatostatin (green) and tachykinin (magenta) (image: Rudi Loesel). Bodian stained brain reveals the PBCh **(D)**, the contralateral projections of *w, x, y, z*, bundles **(E)** and the bistratified architecture of the CB **(F)**. **(G)** Homolateral projections (HP) of the *w, x, y, z* projections between the PB and CB in the fossorial crayfish *Cherax destructor* **(H)** (after Utting et al., 2000). Scale bars: **(C–F)** 100 μm.

With the exception of antennal and antennular movements, and apart from actions by gnathal appendages in feeding, asymmetric appendicular actions may be less common in crustaceans (Marshall and Diebel, 1995). For example, even though male crabs show asymmetric movements of one claw such actions are stereotypic, ritualized signals rather than independent adaptive reactions. Minor elaborations of the CX, such as an additional synaptic layer in the male fiddler crab, denote such sexually dimorphic arrangements (Loesel, unpublished observations), but other than in the CX of stomatopods and possibly in the CX of fast running littoral isopods (Figures 8A–F, see below), there is no clear evidence for a more defined modularity.

CX organization in eumalacostracan crustaceans, as mentioned, generally appears less elaborate when compared with dicondylic insects. In most eumalacostracans, as exemplified by the crayfish *Cherax destructor* (Utting et al., 2000), the PB provides incomplete decussation of axons into a wide spindle-shaped bilayered CB (Figures 8G,H). Although a satellite region comparable to the LAL has been resolved as receiving inputs from the CB, noduli have not yet been documented for any crustacean other than Stomatopoda (see below). As mentioned above, the archaeognathan CX is similarly spindle-shaped, its arrangement suggesting that Archaeognatha is more stemward than is any dicondylic species, an affinity also suggested by the lack of a blood-brain barrier between the archaeognathan circulatory system and retina, implying that this group also shares an important feature of its retinal physiology with marine crustaceans (Shaw and Varney, 1999).

### The Insect-Like Central Complex of Stomatopods

In Malacostraca (and Remipedia; Fanenbruck et al., 2004) the PB is a single span of neuropil extending between the two protocerebral lobes (Sandeman et al., 1988, 1992; Utting et al., 2000; Harzsch and Hansson, 2008), whereas in stomatopods it is distinguished by two extended swellings from each side that meet again at the brain’s mid-line (Figure 3). That this attribute is so far unknown in any other pancrustacean suggests its apomorphic nature. Given that the PB in Dicondylia carries representations of the sensory surround (Heinze and Homberg, 2007; Ritzmann et al., 2008), the functional implications of the organization of the stomatopod bridge will certainly be of future interest. The division of the stomatopod PB into an 8 + 8 modular arrangement suggests that modules may together carry discrete representations of the sensory surround, as demonstrated for Orthoptera, Lepidoptera and Dictyoptera (Heinze and Homberg, 2007; Ritzmann et al., 2008; Heinze and Reppert, 2011).

Another insect-like feature is the manner in which axons extending from the PB decussate into the FB. In decapod malacostracans, exemplified by the crayfish *Cherax destructor* (Figure 8), parallel projections homologous to the *w, x, y, z* bundles of Dicondylia extend ipsilaterally from the PB to the outer layer of the CB to there bifurcate and extend laterally: subunits of the PB are thus represented as overlapping elements within defined domains the CB neuropil (Utting et al., 2000). In stomatopods, on the other hand, axon projections from the PB decussate distal to the FB, as do the *w-, x-, y-*, and *z-bundles* described for the dicondylic brain (Williams, 1975; Boyan et al., 2015), and then overlap each other within the FB itself such that each half of the PB appears to be represented across the whole of the FB.

The stomatopod CB is prominently divided into a distinct upper and lower neuropil, corresponding to the FB and EB found in insects, and as in the insect EB the lower neuropil is correspondingly labeled by anti-DC0 (Figure 5). As in the insect FB, the upper neuropil resolves 4 distinct modules on each side of the midline, again corresponding to the dicondylic arrangement (Figure 4). As in dicondylic insects, prominent tangential neurons in the lower neuropil (the EB) providing large terminal branches are connected by axons to the lateral and median bulb. In *Drosophila* these centers have been shown to encode visual motion information from different segments of each monocular visual field (Seelig and Jayaraman, 2013) and to supply axons to the EB.

The discovery that paired noduli-like neuropils are part of the stomatopod central complex is surprising. These structures, as yet unidentified in any other crustacean, have been suggested as one of the more recently evolved additions to the dicondylic central complex, the proposition being that they may be associated with flight due to their presence in pterygote insects (Homberg, 2008) but not in apterygote Zygentoma (Loesel et al., 2002). They are, however, equally prominent in pterygote species that have an evolved loss of wings, such as the dermapteran *Anisolabis maritima* (Loesel et al., 2002). The presence of noduli in Stomatopoda, which may be unique within crustaceans, allows speculation about their possible association with locomotion. Of all crustaceans, stomatopods may be amongst the most accomplished swimmers, and move with a speed, agility and accuracy not seen in other crustaceans. Are noduli perhaps involved in facilitating such agility? An intriguing finding by Buchanan et al. (2015) is that in *Drosophila*, one synaptic domain in the noduli is involved in the control of handedness during walking. A preference of left vs. right during locomotion may be an important criterion in achieving maneuverability.

One obvious exception to the claim that most eumalacostracans have relatively simple central complexes is Isopoda, particularly in littoral species. *Ligia exotica* (Figure 8) is a fast running eumalacostracan, the CX of which is equipped with a prominent PB and a deep bilayered CB. Its PB consists of two bilateral neuropils connected across the brain’s midline by a system of heterolaterally decussating fibers like those observed in the stomatopod PB. In *L. exotica*, the PB supplies axons to the CB (Strausfeld, 1998, 2012) in a manner reminiscent of the CX of a dicondylic insect; except that in the isopod, axons from one side of the bridge appear to innervate the opposite side of the CB, rather than its entire heterolateral extent (Figure 8).

### Evolutionary Considerations and Correspondences with Dicondylia

**Figure 9:**
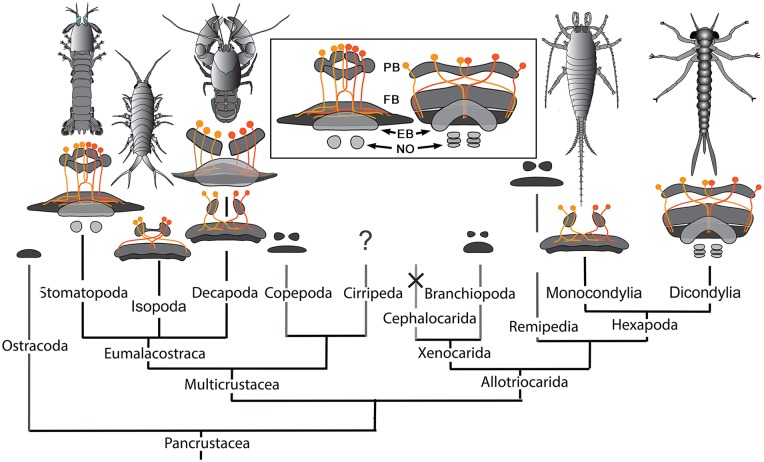
**Central complexes and pancrustacean phylogeny (Oakley et al., 2012).** CX organization in the phyletically distant Stomatopoda and Dicondylia (here represented by an odonate naiad) show close correspondence of their PB, FB, EB, and noduli (NO), and the representation of the PB in the FB by decussating axons (inset). Other CXs in eumalacostracans show simpler arrangements. Decapoda have either homolateral PB-CB projections (as in *C. destructor*) or partially decussating projections, as in Caridea and Dendrobranchiata (examples of species not shown here) that are almost identical to those of monocondylic insects (Strausfeld, 2012). Central complex neuropils, though not their detailed morphologies, have been identified in Branchiopoda (Strausfeld, 2012), Copepoda (Andrew et al., 2012), Remipedia (Fanenbruck et al., 2004), and possibly in Ostracoda. Their presence in cirripede larvae has not been established. There is no evidence for a central complex in Cephalocarida (Stegner and Richter, 2011) where it is assumed to have undergone reduction and loss.

Comparisons across the rather small sample so far investigated suggest that there may be more divergence of CX organization amongst crustacean species than amongst insects, and that organization of CXs in certain basal malacostracans, such as Leptostraca (Strausfeld, 2012; Kenning et al., 2013), might be closest to the ancestral ground pattern. This would be plausible if the most derived crustacean CXs belong to Stomatopoda. Indeed, their dicondylic-like organization (Figure 9 inset) suggests no closer phylogenetic relationship between Stomatopoda and Hexapoda than currently resolved by molecular phylogenetics (Figure 9), which show Eumalacostraca as phyletically distant from the clade comprising Hexapoda and Remipedia (Oakley et al., 2012). But are the dicondylic-like aspects of the stomatopod CX the result of independently evolved convergent elaborations of an ancestral ground pattern? Or might correspondences of the CXs of Dicondylia and Stomatopoda suggest that those lineages alone conserved a far more elaborate ancestral ground pattern than suggested by Leptostraca or any other crustacean? That cerebral organization found in extant eumalacostracans and insects is known to have existed in stem arthropods in the early Cambrian (Ma et al., 2012) admits the possibility that an elaborate central complex may also be as ancient (Strausfeld et al., 2016). Simpler CX arrangements in crown taxa would then reflect an evolutionary history of central complex simplification and in some lineages, such as Cephalocarida, even complete loss.

What are the distinctive characters of malacostracan CXs that sets them apart, and which may impact considerations about functional commonalities? The proposition that CXs may be computationally equivalent in mediating compass headings and path integration (Webb and Wystrach, 2016) still requires reflection on the diversity of CX arrangements across pancrustaceans. In dicondylic insects, for example, major inputs to layers of the FB originate from the medial lobes of the protocerebrum, a complex neuropil that is supplied by, if not all, then the majority of output neurons from the mushroom body lobes (Li and Strausfeld, 1997, 1999; Phillips-Portillo and Strausfeld, 2012; Wolff and Strausfeld, 2016). If eumalacostracans generally do not possess these iconic centers then such circuits might be entirely absent. Indeed, the lack of prominent multistratified FBs in crustaceans (and in archaeognathans, which lack mushroom bodies) suggests a major functional difference from their dicondylic counterparts, stomatopods again being the exception. In Dicondylia, the multiple strata that define the FB reflect the elaboration and identities of its inputs, particularly from the protocerebral lobes, as well as arrangements of numerous species of peptidergic neurons (Herbert et al., 2010; Kahsai and Winther, 2011) certain of which are implicated in the modulation of locomotory activity (Kahsai et al., 2010).

Of the many divergent CX arrangements across Dicondylia, one is particularly notable. This relates to the representation of appendicular control. In Dicondylia, modular organization of the CX’s FB and EB appear to directly bear on appendicular versatility, such as required for directional change during walking, obstacle avoidance, climbing and even predation. The precision of modular organization in the FB, allowing the recognition of 4 + 4 discrete modules on each side of the midline, relates to the amount of overlap between dendrites and collaterals belonging to neurons in adjacent modules, there being the least overlap in those species with the most refined appendicular dexterity (Strausfeld and Hirth, 2013). Surveys across eumalacostracan central complexes have not resolved comparable distinctions. The exception is in fossorial crayfish, in which the CB is divided into 4 + 4 modules (Utting et al., 2000), littoral isopods, where the foliated CB comprises 8 + 8 modules, and stomatopods where immunocytology clearly identifies eight subdivisions across the FB (Figure 4).

Differences of organization amongst homologous neurons reflect specific differences of synaptic arrangements within homologous circuits. Such differences may provide important avenues towards interpreting the significance of CX neuroanatomy with respect to motor repertoires and the ecological constraints in which they are elicited. It is not just in insects that divergent modifications of the ancestral ground pattern may reveal functional attributes. For example, despite the relative few detailed studies of eumalacostracan brains, it is notable that both the morphology of the PB, and the organization of the *w, x, y, z* fibers from it, can differ substantially. The relatively simple unistratified PB of the crayfish *Cherax destructor* (Utting et al., 2000) occupies a span broader than that of the more elaborate stomatopod PB. However, in *C. destructor* the *w, x, y, z* tracts do not decussate as they do in Stomatopoda and Dicondylia (Figures 3, 8) but project directly into the CB beneath. In littoral isopods, such as *Ligia exotica*, *w, x, y, z* tracts project from each half of the PB into the contralateral half of the CB rather than distributing across the whole of CB (Figure 8). Such differences of representation in the PB would suggest important differences of homologous computational circuits and it is of considerable interest that in Dicondylia only some classes of neurons from the PB do indeed decussate. As described from *Drosophila* and *Schistocerca*, certain modular neurons from each of half the PB map across the whole FB, as in Stomatopoda. These contrast with a class of PB neurons in Dicondylia, the axons of which extend directly to the FB without any decussation, only to undergo heterolateral projection from the FB into the LAL (Hanesch et al., 1989; Heinze and Homberg, 2008).

### The Central Complex and Efferent Sensory-Motor Convergence

In conclusion, our still preliminary studies of the stomatopod central complex suggest numerous similarities with CXs of dicondylic insects (for a summary of shared features see Table 2). Across eumalacostracans, and mostly likely in other crustacean groups as well, antennules, antennae and gnathal appendages can all show lateralized as well as bilateral coordination. For example, fossorial decapods dig burrows and walk about; littoral isopods run rapidly, and even jump across gaps. Nevertheless, certain appendicular actions of stomatopods do stand apart. One is cleaning actions by the maxillipeds in maintaining debris-free retinal surfaces. Another is the range of actions executed by the large antennal plates or scales that appear to play a crucial role in swimming, possibly serving a dual function in mechanosensory input. And lastly, one of the most interesting actions is the extraordinary range of independent movements carried out by the eyestalks.

**Table 2:** **Summary of central complex features found across groups of crustaceans, monocondylic and dicondylic insects**.

	Stomatopoda	“Other” crustaceans	“Dexterous” dicondylic insects	“Non-dexterous” dicondylic insects	Monocondylic insect (Archaeognatha)
Protocerebral bridge (PB)	Prominent	Varied: in some species small inconspicuous and lateralized.	Prominent	Prominent	Small, lateralized
Complete decussation of axons from PB	Yes	“Incomplete” decussation: PB represent mainly in contralateral half of FB. (see Figure 8); or, homolateral PB-CB projection.	Yes: each side of the PB represented across entire FB	Yes, each side of the PB represented across entire FB	“Incomplete” decussation: PB represent mainly in contralateral half of FB.
Central body bilayered or multi component	Central body (CB) divided into two discrete stratified neuropils, the FB and EB	At least two layers resolvable with certain antibodies	Central body divided into two discrete stratified neuropils, the FB and EB	Central body divided into two discrete stratified neuropils, the FB and EB	At least two layers resolvable with certain antibodies
Central body shape	Broad tapering upper division, narrower lower division	Usually spindle-shaped	Fan-shaped upper level, arched- to ellipsoid shaped body lower level	Fan-shaped upper level, arched- to ellipsoid- shaped body lower level	Spindle-shaped
Prominent modules in the fan-shaped body	Yes	Rarely	Yes	No	No
Noduli present	Yes	None identified	Yes	Yes	None identified

As introduced at the beginning of this article, the highly modular arrangement of the stomatopod FB combined with refined appendicular dexterity lends support to the proposition that the CX, as a recipient of inputs from both eyestalks (Figures 6, 7), is a likely candidate for the control of their conjoint movements. Although no studies on insect CXs show a role in action selection by head appendages, the anatomical and physiological organization of crustacean eyestalks conform to a sensory and motor ground pattern typifying jointed appendages, such as the legs. Eumalacostracan eyestalks usually comprise three articles, albeit fused: proximal, medial and distal segments, the last surmounted by the eye. Eyestalks are equipped with muscles providing coordinated rotational and translator movements in response to visual and gravitational stimuli (Mellon, 1977). Thus, as do legs, eyestalks perform discrete behavioral actions in response to specific multisensory stimuli. In stomatopods, the two eyestalks can switch from independent movements to conjoint scans of potential prey using saccadic movements for acquisitive vision (Marshall et al., 2014). Such responses are comparable to visually-driven leg movements of mantispid Neuroptera during prey tracking (Kral et al., 2000). The appendicular nature of the eyestalk was well known to 19th Century zoologists who demonstrated that an articulated appendage is regenerated in lieu of an amputated eyestalk (Milne-Edwards, 1864). And in insects, suppressing genes that determine the development of a compound eye results in the default development of an articulated appendage in place of the eye (Kumar and Moses, 2001).

In addition to their appendicular nature, neuroanatomical evidence shows that the two eyestalks provide numerous efferent axons that converge at the stomatopod CX. The existence of these pathways (Figures 6, 7), combined with the highly modular arrangement of the stomatopod FB, strengthens the proposition that this locus of efferent convergence may be pivotal to eyestalk action selection. Furthermore, preliminary electrophysiological recordings using sharp extracellular electrodes coated with fluorescent dye demonstrate that it is neurons in the stomatopod CX that vigorously respond to visual movements, being activated immediately prior to movements of the two eyes (N. Lessios, unpublished data).

**Figure 10:**
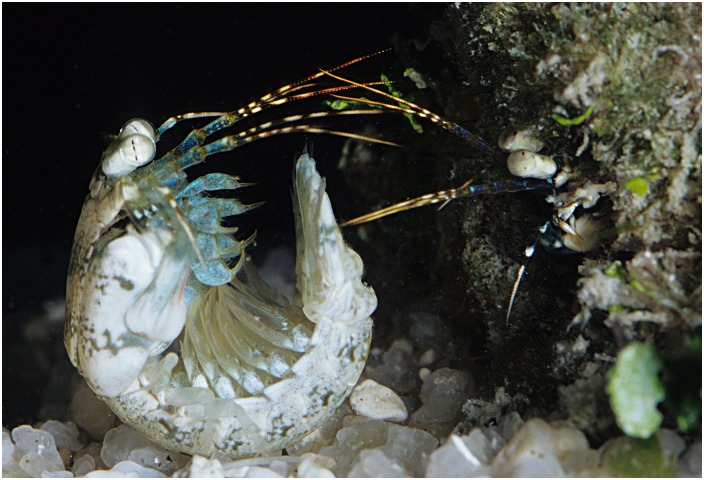
**Behavioral actions in stomatopods.** Stomatopods frequently compete over burrows in coral reef substrate. *Haptosquilla trispinosa* (shown here) also meet for potential mating and both activities may be hazardous, hence the approach of the intruder/suitor using the telson as armor. During these encounters, sensory structures such as the antennae, antennules, antennal scales and eyes (for clearer view, see Figure 2) are pointed in a forward position and actively gather information through both independent and conjoint movements. Image: Roy Caldwell.

Doubtlessly there are other behaviors that are under control of action-selecting circuits. Figure 10 shows an example of an encounter between two stomatopods, manifesting some of a range of behavioral actions. However, whereas these are not unique to this group of eumalacostracans, independent eye movements are unparalleled except in two other taxa both of which are vertebrates. One is chameleons, in which each eye functions independently of the other and, as the authors suggest, each half of the brain is likely associated with homolateral oculomotor and visual processing (Tauber and Atkin, 1967). The other is the sandlance *Limnichthyes fasciatus*, a teleost that like the chameleon and stomatopod employs ballistic strikes to capture prey (Fritsches and Marshall, 1999). The ability of the stomatopod to switch from independent optokinetic nystagmus and visual pursuit to tight collaboration of the two eyes during fixation and targeting provides a fascinating behavior that demands the identification of neural circuits mediating its orchestration. Thus far, neuroanatomical observations suggest that the CX might be the most likely candidate.

## Ethics Statement

This study was carried out in accordance with the recommendations of the Australian Code of Practice for the Care and Use of Animals for Scientific Purposes, National Health and Medical Research Council. The protocol was approved by the University of Queensland Animal Ethics Committee (AEC).

## Author Contributions

NJS, HHT, GHW and JM designed the study, NJS, HHT and GHW collected the data; NJS and HHT made the figures; NJS, HHT, GHW and JM wrote the manuscript.

## Funding

This work was supported by a grant from the U.S. Air Force Research Laboratory (FA86511010001) and the Center for Insect Science, University of Arizona to NJS, grants to JM from the Asian Office of Aerospace Research and Development (AOARD-12-4063) and the Australian Research Council (FL140100197), and a grant to HHT from the Lizard Island Research Foundation, a Doctoral Fellowship (2013) from the Lizard Island Research Station, a facility of the Australian Museum.

## Conflict of Interest Statement

The authors declare that the research was conducted in the absence of any commercial or financial relationships that could be construed as a potential conflict of interest. The reviewer MB and handling Editor declared their shared affiliation, and the handling Editor states that the process nevertheless met the standards of a fair and objective review.
